# Transcriptomic Alterations in Spliceosome Components in Advanced Heart Failure: Status of Cardiac-Specific Alternative Splicing Factors

**DOI:** 10.3390/ijms25179590

**Published:** 2024-09-04

**Authors:** Isaac Giménez-Escamilla, Lorena Pérez-Carrillo, Irene González-Torrent, Marta Delgado-Arija, Carlota Benedicto, Manuel Portolés, Estefanía Tarazón, Esther Roselló-Lletí

**Affiliations:** 1 Clinical and Translational Research in Cardiology Unit, Health Research Institute Hospital La Fe (IIS La Fe), Avd. Fernando Abril Martorell 106, 46026 Valencia, Spain; isaac_gimenez@iislafe.es (I.G.-E.); lorena_perezc@iislafe.es (L.P.-C.); irene_gonzalez@iislafe.es (I.G.-T.); marta_delgado@externos.iislafe.es (M.D.-A.); carlota_benedicto@iislafe.es (C.B.); drmanuelportoles@gmail.com (M.P.); 2 Center for Biomedical Research Network on Cardiovascular Diseases (CIBERCV), Avd. Monforte de Lemos 3-5, 28029 Madrid, Spain

**Keywords:** spliceosome, heart failure, ischaemic cardiomyopathy, dilated cardiomyopathy, alternative splicing

## Abstract

Heart failure (HF) is associated with global changes in gene expression. Alternative mRNA splicing (AS) is a key regulatory mechanism underlying these changes. However, the whole status of molecules involved in the splicing process in human HF is unknown. Therefore, we analysed the spliceosome transcriptome in cardiac tissue (n = 36) from control subjects and HF patients (with ischaemic (ICM) and dilated (DCM) cardiomyopathies) using RNA-seq. We found greater deregulation of spliceosome machinery in ICM. Specifically, we showed widespread upregulation of the E and C complex components, highlighting an increase in *SNRPD2* (FC = 1.35, *p* < 0.05) and *DHX35* (FC = 1.34, *p* < 0.001) mRNA levels. In contrast, we observed generalised downregulation of the A complex and cardiac-specific AS factors, such as the multifunctional protein *PCBP2* (FC = −1.29, *p* < 0.001) and the RNA binding proteins *QKI* (FC = −1.35, *p* < 0.01). In addition, we found a relationship between *SNPRD2* (an E complex component) and the left ventricular mass index in ICM patients (r = 0.779; *p* < 0.01). On the other hand, we observed the specific underexpression of DDX46 (FC = −1.29), *RBM17* (FC = −1.33), *SDE2* (FC = −1.35) and *RBFOX1* (FC = −1.33), *p* < 0.05, in DCM patients. Therefore, these aetiology-related alterations may indicate the differential involvement of the splicing process in the development of ICM and DCM.

## 1. Introduction

Heart failure (HF) is a clinical syndrome with symptoms or signs caused by a structural or functional cardiac abnormality, corroborated by elevated natriuretic peptide levels or objective evidence of cardiogenic pulmonary or systemic congestion [1]. The pathogenesis of HF has often been categorised in clinical practice as ischaemic cardiomyopathy (ICM) or dilated cardiomyopathy (DCM) [2]; both constitute approximately 80% of advanced HF diagnoses [3]. Prior research has demonstrated that HF is linked to gene expression alterations disrupting various cellular functions and structures, such as the cardiomyocyte cytoskeleton [4], natriuretic peptides pathways [5] or mitochondrial dysfunction [6,7]. Alternative splicing (AS) is a key regulatory mechanism underlying gene expression changes [8]. The advancement of next-generation sequencing has allowed us to identify that 95% of human genes undergo AS [9].

AS is the molecular process by which non-coding intervening sequences (introns) are removed and some neighbouring coding regions (exons) may be included in or excluded from the final RNA product of the gene, resulting in various forms of mature mRNA for protein biosynthesis [10]. AS is carried out by the spliceosome, a multi-megadalton RNA protein molecular machine composed of small nuclear ribonucleoproteins (snRNPs) and a plethora of auxiliary non-snRNPs [11,12]. The spliceosome functions by following a stepwise assembly involving a sequence of different complexes [10]. Changes in the splicing process can produce numerous pathologies including cardiovascular diseases, whether due to modifications in the spliceosome-binding sites or the participating factors [13]. Multiple splicing factors have been identified as necessary for cardiac development. Incorrect protein isoforms can alter the functioning of the sarcomere, ion channels and cell signalling molecules. Additionally, the absence of non-snRNP protein families can cause human cardiovascular diseases, such as HF [11].

Therefore, AS is a key mechanism involved in gene expression regulation, and alterations in its components have been related to the development of different diseases [14,15,16,17]. Nevertheless, a whole study of the components of the spliceosome and the AS factors related to human HF has not yet been performed. Hence, we carried out a transcriptomic study to know the status of the different spliceosome components and cardiac-specific AS factors in patients with HF of ICM and DCM aetiologies.

## 2. Results

### 2.1. Clinical Characteristics of Patients

The patients’ clinical and echocardiographic characteristics and treatment are summarised in Table 1. The patients had a mean age of 53 ± 10 years, and most were men (96%). They belonged to classes III–IV of the New York Heart Association (NYHA) functional classification and presented altered values in echocardiographic parameters (the left ventricular ejection fraction (LVEF), left ventricular end-systolic (LVESD) and end-diastolic (LVEDD) diameters and the left ventricular mass index (LVMI)). Comorbidities, including hypertension and diabetes mellitus, were identified. The control (CNT) group consisted mainly of men (80%) with a mean age of 47 ± 16 years.

### 2.2. mRNA Expression of Spliceosome Components in Advanced Heart Failure Patients

**Figure 1:**
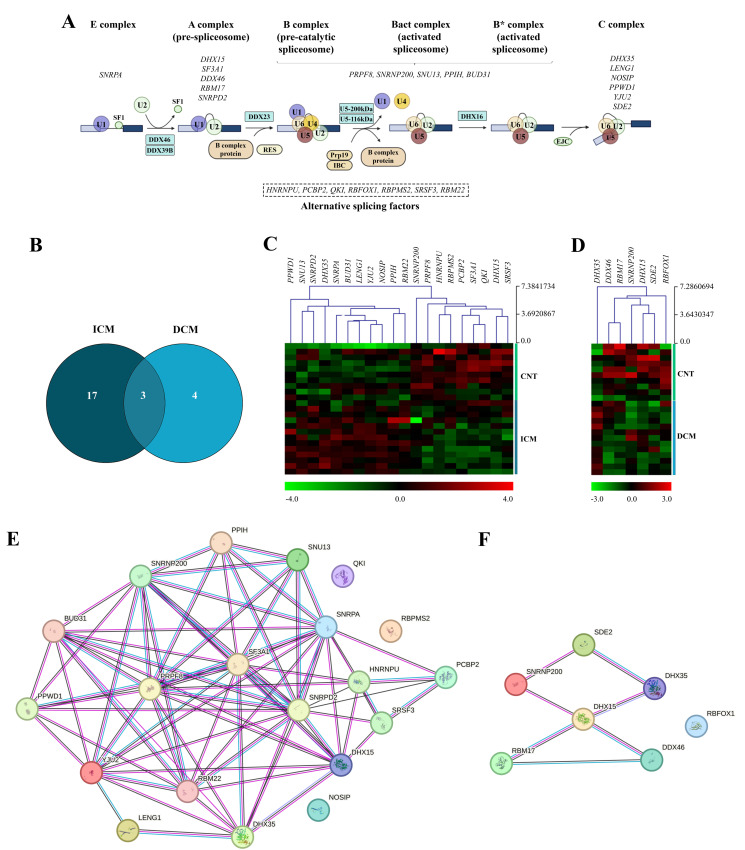
Spliceosome components status in advanced heart failure patients. (**A**) Sequential formation of spliceosome complexes: E complex is formed upon U1 snRNP’s recognition of 5′ splicing site. Once U2 snRNP replaces SF1, it transforms into the A complex. Subsequently, the union of U4, U5 and U6 snRNPs gives rise to inactive complex B. A major compositional and conformational remodelling forms Bact and B* complexes, becoming catalytically active. After the first transesterification reaction, the C complex is formed. The altered molecules are listed for each complex alongside the alternative splicing factors (dotted box). (**B**) Venn diagram highlighting similarities and differences in genes of major spliceosome, with altered expression between ischaemic (ICM) and dilated (DCM) cardiomyopathies. (**C**,**D**) Heat map summarising mRNA expression levels of spliceosome components from control (CNT) subjects and ICM and DCM patients, respectively. Each row represents a sample, and each column represents an altered mRNA. Colours depict the relative expression level of each molecule, with green being the lowest and red the highest. (**E**,**F**) Schematic string interaction analysis showing protein–protein interactions of spliceosome components and heart-related splicing factors differentially expressed in ICM and DCM, respectively. Edges represent protein–protein associations including known interactions from curated databases (light blue), experimentally determined (pink) and co-expression (black).

We focused on studying the genes involved in the major spliceosome machinery (Table S1) and heart-tissue-specific AS factors [15,18,19] (Table S2, Figure 1A). The results showed a deregulation of the spliceosome machinery that differed between the aetiologies, obtaining a greater dysregulation in ICM patients compared with DCM patients, as shown in the Venn diagram (Figure 1B). Hierarchical clustering of these altered genes showed a marked difference between ICM and DCM patients and the CNT group (Figure 1C,D, respectively). Furthermore, Figure 1E,F shows a protein–protein interaction network for the altered genes in ICM and DCM, respectively.

#### 2.2.1. Alterations in E, A, B and C Complexes

**Figure 2:**
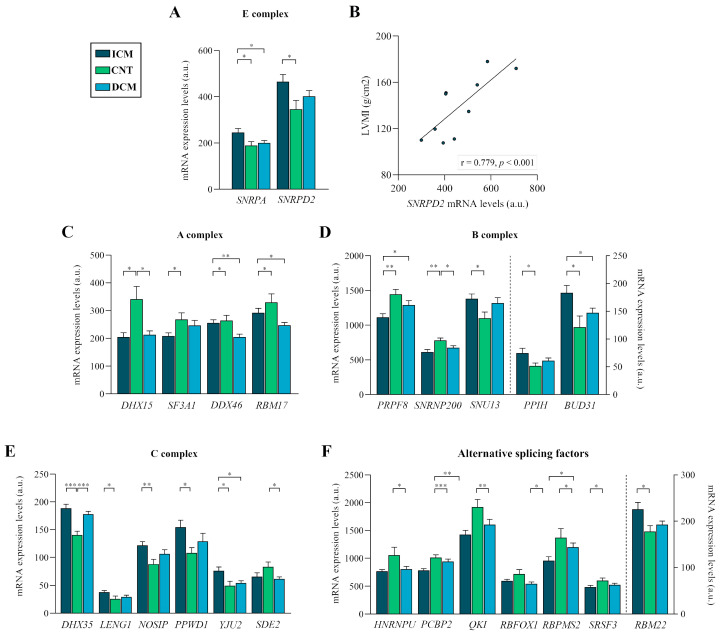
Altered mRNA expression levels of spliceosome components and splicing factors in advanced heart failure patients. (**A**) mRNA expression levels of E complex components. (**B**) Relationship between mRNA relative expression levels of *SNRPD2* and left ventricular mass index (LVMI) in ischaemic cardiomyopathy patients. (**C**) mRNA expression levels of A complex components. (**D**) mRNA expression levels of B complex components. (**E**) mRNA expression levels of C complex components. (**F**) mRNA expression levels of alternative splicing factors. Data are presented as the mean ± SEM; a.u., arbitrary units. Statistical differences found between ischaemic cardiomyopathy, control and dilated cardiomyopathy groups: * *p* < 0.05, ** *p* < 0.01 and *** *p* < 0.001.

The spliceosome assembly process entails the stepwise recruitment of different complexes (Figure 1A). During assembly, the U1 snRNP recognises the 5′ splicing site (E complex) [10]. In this complex, the ICM group showed an overexpression compared to the CNT group in *SNRPA* (FC = 1.30, *p* < 0.05) and *SNRPD2* (FC = 1.34, *p* < 0.05) (Figure 2A). In addition, *SNRPD2* showed a positive correlation with the LVMI (r = 0.779, *p* < 0.001) (Figure 2B), the established echocardiographic parameter of cardiac remodelling.

Next, the A complex is formed after the replacement of SF1 by U2 snRNP [10] (Figure 1A). All altered molecules exhibited decreased expression levels in patients compared to the CNT group. The DCM group showed underexpression in *DHX15* (FC = −1.60, *p* < 0.05), *DDX46* (FC = −1.29, *p* < 0.05) and *RBM17* (DCM: FC = −1.33, *p* < 0.05); and the ICM group showed underexpression in *DHX15* (FC = −1.66, *p* < 0.05) and *SF3A1* (FC = −1.29, *p* < 0.05) (Figure 2C).

The union of the U4, U5 and U6 snRNPs generates a pre-formed tri-snRNP, giving rise to the B complex [10] (Figure 1A). Our data showed a general deregulation in ICM patients compared to the CNT group (Figure 2D), specifically an underexpression of *PRPF8* (FC = −1.30, *p* < 0.01) and *SNRNP200* (FC = −1.27, *p* < 0.01); and an overexpression of *SNU13* (FC = 1.26, *p* < 0.05), *PPIH* (FC = 1.45, *p* < 0.05) and *BUD31* (FC = 1.51, *p* < 0.05). In addition, the DCM group showed an underexpression of *SNRNP200* (FC = −1.16, *p* < 0.05) compared to the CNT group (Figure 2D).

The two-step transesterification mechanism of pre-mRNA splicing by the major spliceosome undergoes different conformations as elements are added. In this sense, we found alterations related to the components recruited in the last complex involved in the reactions [10] (Figure 1A). We found a general upregulation in the C complex components in the ICM group compared to the CNT group: *DHX35* (FC = 1.34, *p* < 0.001), *LENG1* (FC = 1.47, *p* < 0.05), *NOSIP* (FC = 1.38, *p* < 0.01), *PPWD1* (FC = 1.43, *p* < 0.05) and *YJU2* (FC = 1.53, *p* < 0.05) (Figure 2E). *DHX35* maintained overexpression in the DCM group (FC = 1.27, *p* < 0.001) and *SDE2* was the only downregulated C complex component in this group compared to controls (FC = −1.35, *p* < 0.05) (Figure 2E).

#### 2.2.2. Cardiac-Specific AS Factors

AS factors regulate the expression of protein isoforms to adapt cardiac function to the changing demands during development and disease [11] (Figure 1A). Specifically, our results showed a general downregulation in the ICM group compared to the CNT group of the nuclear ribonucleoprotein *HNRNPU* (FC = −1.37, *p* < 0.05), the multifunctional protein *PCBP2* (FC = −1.29, *p* < 0.001), the RNA-binding proteins *QKI* (FC = −1.35, *p* < 0.01) and *RBPMS2* (FC = −1.43, *p* < 0.05) and the splicing factor *SRSF3* (FC = −1.24, *p* < 0.05), as well as the RNA-binding protein *RBFOX1* in the DCM group (FC = −1.33, *p* < 0.05) (Figure 2F). Among the molecules analysed, only *RBM22*, an RNA-binding protein, was overexpressed in the ICM group compared to the CNT group (FC = 1.27, *p* < 0.05) (Figure 2F).

## 3. Discussion

RNA splicing has emerged as a crucial process implicated in the mechanisms underlying cardiovascular diseases. Alterations in the components of the spliceosome and the different heart-related AS factors can disrupt the normal architecture and homeostasis of the heart [11,13,20]. In this study, we showed molecular alterations in key components of the sequential conformation complexes involved in AS, as well as in cardiac-specific AS regulatory factors in HF patients. We observed a differential expression pattern in these patients, demonstrating greater dysregulation in the ICM group than in the DCM group. Since cardiomyopathies impact the function and structure of the myocardium in a diverse manner [21], these aetiology-related alterations could indicate the differential participation of the splicing process in the development of both aetiologies.

The function of the spliceosome depends on the recognition of intronic boundaries, with the U1 snRNP being responsible for the initial binding to the 5′ splicing site of the pre-mRNA [22]. Thus, the formation of the E complex is the earliest event that initiates the splicing process [23]. Small nuclear ribonucleoprotein polypeptide A (SNRPA) is a specific molecule of the U1 snRNP, and our results showed an overexpression of its mRNA levels in ICM patients. In a previous study, SNRPA was found to bind directly to the *STAT5B* 3′ UTR and facilitate its alternative polyadenylation (a process coordinated with AS) [24], switching when T-cell receptor signalling is active [25], thus resulting in increased mRNA stability and enhanced STAT5B protein expression involved in T-cell activation and apoptosis, characteristic processes in ICM. Taking all together, it could be possible that also in ischaemic conditions, where activated T lymphocytes are essential drivers [26], SNRPA upregulation leads to alternative polyadenylation switching of *STAT5B*. Likewise, it has been observed that overexpression of SNRPD2, an Sm protein forming part of the U1 snRNP structure, can also promote the proximal alternative polyadenylation sites at the transcriptome level [27]. In this study, we found that *SNRPD2* upregulation in ischaemic hearts was related to a greater LVMI, and in this sense an appropriate next step might be to delve deeper into the role of this molecule in the cardiac remodelling and fibrosis processes, characteristic entities with prognostic implications in this pathology.

The pre-spliceosome (A complex) is formed when the E complex recruits the U2 snRNP. This complex exhibited a widespread downregulation of its components in both aetiologies. Deficiency of *DHX15*, an ATP-dependent RNA helicase that dissociates the spliceosome molecules [28,29], has been linked to impairing endothelial energy metabolism impairment, specifically with the alteration of mitochondrial complexes, which results in lower intracellular ATP production [28]. We have previously shown a deregulation of the oxidative phosphorylation system, which produces most of the ATP consumed by the heart in the inner mitochondrial membrane, in ICM patients [6]. These results could be related to the decrease in *DHX15* expression in ICM patients observed in this study. On the other hand, the quality control of the initial interaction between the U2 snRNP and an intron performed by DHX15 could be performed in conjunction with DDX46 [29]. Both molecules are downregulated in DCM, so this quality control could be a critical point in the development and progression of DCM. Finally, we observed a decrease in the expression of *SF3A1*, which has been identified as an essential splicing factor for direct cardiac reprogramming [30]. The heart is one of the least regenerative organs in the body; cardiomyocyte division or generation from progenitor cells probably occurs in the human heart, but it is a very slow process [31]. Both can contribute to advanced HF since cardiomyocyte deficiency underlies most causes of HF.

The B complex comprises the pre-catalytic (B complex) and activated (Bact complex and B* complex) spliceosome. We observed several alterations of B complex components in ICM patients. Specifically, we found a downregulation in pre-mRNA processing factor 8 (*PRPF8*) and small nuclear ribonucleoprotein 200 kDa (*SNRNP200*); both molecules are essential constituents of the U5 snRNP. PRPF8 is a regulator of SNRNP200 helicase function [32], and the downregulation of both molecules is related to defects in mitophagy [33]. Moreover, PRPF8 has been reported to induce mitophagy in response to hypoxia in retinitis pigmentosa [34]. ICM is mainly caused by long-term ischaemia/hypoxia, which causes mitochondrial damage. Under stress conditions, mitophagy clears damaged mitochondria to maintain cellular health, but excessive or insufficient mitophagy can lead to cell damage and death [35]. Although the specific functions of PRPF8 and SNRNP200 in HF are unknown, they could be involved in mitochondrial homeostasis in response to stress. On the other hand, we observed an upregulation of peptidyl-prolyl cis–trans isomerase H (*PPIH*) and BUD31 homolog (*BUD31*) in patients with advanced ICM. PPIH is associated with the tri-snRNP (U4/U6.U5), while BUD31 helps stabilise the 5′ stem loop and downstream U6 snRNA sequences. The overexpression of both molecules at the mRNA level correlates with poor patient outcomes and advanced disease stages in several solid cancers [36,37]. However, the status of these molecules in different HF stages has not been studied but could contribute to the progression of the disease.

The C complex presents the association of several protein components and catalysing exon ligation. We described a generalised upregulation of this complex in ICM patients. A stand-out is the helicase DEAH-box adenosine triphosphatases (ATPases) (*DHX35*) dysregulation in both aetiologies, which has not been previously described in cardiovascular diseases. DHX35 plays a crucial role in the spliceosome-mediated excision of pre-mRNA introns, and a decrease in its levels results in reduced splicing efficiency [38,39]. However, detailed mechanistic insights into how these components function as a molecular machine remain scarce. Thus, a better understanding of the molecular mechanism underlying translocation might help to know the larger dynamic events of the spliceosome in HF. Our group has previously described an increase in *NOSIP* levels in ICM patients related to the inhibition of nitric oxide synthase 1 (NOS1) activity, a major modulator of cardiac function, despite *NOS1* upregulation by the induction of its translocation to the sarcolemma [40,41]. NOSIP has also been described as a spliceosomal protein that limits splicing errors, and its absence is related to splicing efficiency [38,42]. Thus, NOSIP overexpression may be involved in kinetic proofreading mechanisms, thereby also modulating cardiac function.

Additionally, cardiac-specific AS factors have been described and are involved in generating specialised protein isoforms that allow the heart to adapt during development and disease. Since the discovery of RBM20’s role as a heart AS factor, which regulates a network of splicing events in genes related to sarcomere structure and calcium handling and whose alterations are involved in DCM development [43], many investigations have focused on the study of AS factors in the heart [44]. We observed in ICM patients an overexpression of *RBM22*, a family member of RBM20, which has not yet been studied in the context of HF. However, a relevant role of RBM22 has been described in the translocation of the calcium-binding protein ALG-2 [45] that is not altered under stress conditions [46], making RBM22 a key molecule in calcium homeostasis. In contrast, the other altered AS factors exhibited reduced expressions in HF. QKI has been described as an anti-apoptotic protein in ischaemia/reperfusion assays in neonatal cardiomyocytes and adult rat hearts that is essential to normal cardiogenesis and cardiac function [47]. Moreover, QKI regulates the AS of more than 1000 genes, including sarcomeres and cytoskeletal components. *QKI* deletion has been linked to the development of HF associated with severe sarcomere disruption. This makes QKI a muscle-specific AS factor that regulates the contractile function of cardiomyocytes [48,49]. In this study, we described for the first time the underexpression of this molecule in human cardiac tissue from patients with advanced HF. Furthermore, our data confirm the previously described downregulation of *PCBP2* in advanced failing human hearts [50] and the reduction in *SRSF3* expression after myocardial infarction related to severe systolic dysfunction and death, suggesting a possible role in cardiac homeostasis via sarcomere organisation and calcium handling [51]. Similarly, reduction in *RBPMS2* expression, which we observed in ICM patients, has also been observed to cause sarcomere organisation, calcium handling and cardiac function defects [52]. We also described a downregulation of the mRNA of heterogeneous nuclear ribonucleoprotein U (*HNRNPU*). Previous studies have demonstrated the essential role of hnRNP U in postnatal heart development and function and AS regulation. Therefore, mice lacking hnRNP U exhibit a rapid progression of HF (lethal DCM with a similar phenotype) and display numerous defects in cardiac pre-mRNA splicing [53]. Furthermore, its absence is related to heart regeneration prevention in mice after myocardial infarction regulating cardiomyocyte proliferation and reparative angiogenesis to control the regenerative window in the heart [54]. RBFOX1 was the only heart AS factor altered in DCM patients, consistent with our results; a downregulation in RBFOX1 has been previously described in failing human and mouse hearts. Deficiency in the heart of RBFOX1, being an important player in transcriptome reprogramming in HF [55], promotes pressure-overload-induced cardiac hypertrophy [56].

Mutations in the splicing machinery can result in a range of human diseases and disorders [57], as well as the deregulation of its components [58], similarly to what we observed with changes in gene expression in HF. A key factor in the research of these diseases is the identification of mutations or alterations in the expression of splicing factors, their effect on the transcriptome and possible pharmacological targets. Thus, U1 snRNA mutations have been associated with medulloblastomas, U2 SF3B1 mutations with blood cancers such as myelodysplasia or lymphocytic leukaemia, U5 Prp8 mutations with retinitis pigmentosa [57,59] and QKI downregulation with lung cancer [58]. Knowing these changes has allowed the development of different treatment approaches directed in some way toward influencing splicing outcomes by direct interactions with splicing factors (general inhibitors of the spliceosome that alter, or prevent, splicing of hundreds or thousands of transcripts) or design molecules that change alternative splicing patterns of certain RNAs [57]. So global changes in the splicing program of a cell can be useful for triggering apoptosis, while correcting the splicing of just a single gene can restore protein and cell function. Hence, knowledge of the alterations in the expression of the splicing machinery in HF may be useful for the development of new therapies aimed at restoring protein and cell function.

The wide variation in subjects as well as their treatment, some of which may affect the results, is a major limitation of research that focuses on cardiac tissues from end-stage human HF. Additionally, the samples analysed were mainly from men, due to the higher prevalence of ICM, in accordance with data published in the Heart Disease and Stroke Statistical Update of the American Heart Association (AHA) [60]. Our research population had a homogeneous aetiology, and all the patients examined were receiving medical treatment following the recommendations of the European Society of Cardiology [21]. On the other hand, although RNA-seq expression levels correlate well with RT-qPCR-based quantification, another limitation is the lack of validation of expression profiles using this technique.

Our results show relevant alterations in the spliceosome machinery and cardiac-specific AS factors in HF, finding greater dysregulation in ICM patients than in DCM patients. These changes could indicate a differential involvement in their development and provide knowledge to understand the specific molecular mechanisms contributing to HF aetiologies. We observed a widespread upregulation of the E and C complex components, contrasting with the generalised downregulation of the A complex and cardiac-specific splicing factors. In addition, we showed a relationship between *SNPRD2* and cardiac remodelling in ICM patients.

## 4. Materials and Methods

### 4.1. Tissue Sample Collection

**Figure 3:**
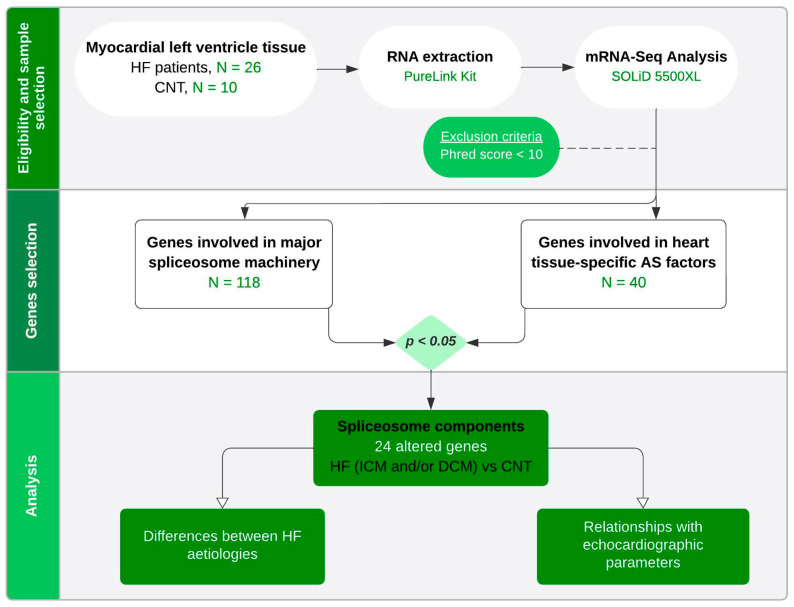
Gene expression study flowchart. Summary of analysis approach and findings on gene expression analysis of the spliceosome components. HF, heart failure; CNT, controls; AS, alternative splicing; ICM, ischaemic cardiomyopathy; DCM, dilated cardiomyopathy.

A total of 36 myocardial left ventricular tissue samples were collected from the ventricle’s apex of explanted human hearts belonging to CNT (n = 10) donors and patients diagnosed with ICM (n = 13) and DCM (n = 13) receiving heart transplantation (Figure 3). After extraction, the samples were maintained in 0.9% NaCl at 4 °C for a maximum of 4.4 ± 3 h after loss of coronary circulation and stored at −80 °C until use.

Clinical history, electrocardiogram, echocardiographic parameters, hemodynamic studies and coronary angiography data were available for all patients. DCM was diagnosed when patients had a LVEF < 40% with a LVEDD > 55 mm. History of documented acute myocardial infarction episodes, the existence of normal contractility segments co-existing with other akinetic or dyskinetic segments shown using echocardiography and the emergence of myocardial necrosis or ischaemia signs on electrocardiography were used to diagnose ICM. Every patient was given medical treatment in accordance with the standards set out by the European Society of Cardiology [21] and was functionally categorised using the NYHA functional criteria.

The CNT samples were collected from non-diseased hearts that were ineligible for transplantation because of blood incompatibility or surgical reasons. Motor vehicle accidents or cerebrovascular incidents were the donors’ reasons of death. Every one of the CNT hearts had a normal LVEF (≥50%). In compliance with the Spanish Organic Law on Data Protection 15/1999, the only available data were age and gender.

### 4.2. RNA Extraction and Integrity

The cardiac samples (CNT, n = 10; ICM n = 13; DCM, n = 13) were homogenised with TRIzol reagent in a TissueLyser LT (Qiagen, Hilden, Germany). RNA extractions were conducted using a PureLink™ Kit in accordance with the instructions provided by the manufacturer (Ambion Life Technologies, Vilnus, Lithuania). The quantification of RNA was performed using a NanoDrop1000 spectrophotometer (Thermo Fisher Scientific, Waltham, MA, USA), while the purity and integrity of the RNA samples were assessed using an Agilent 2100 Bioanalyser with an RNA 6000 Nano LabChip kit (Agilent Technologies, Santa Clara, CA, USA). All samples had a 260/280 ratio greater than 2.0 and an RNA integrity number of 9 or above.

### 4.3. mRNA-Seq Analysis

Using a MicroPoly (A) Purist Kit™ (Ambion Life Technologies, Vilnus, Lithuania), 25 micrograms of total RNA was used to isolate the polyA-RNA. Following the manufacturer’s instructions (Ambion Life Technologies, Vilnus, Lithuania), whole-transcriptome libraries generated from the total polyA-RNA samples were sequenced using the SOLiD 5500XL platform. The Qubit 2.0 Fluorometer (Invitrogen, Waltham, MA, USA) was used to quantify the amplified cDNA, and the Bioanalyser 2100 DNA 1000 kit (Agilent Technologies, Santa Clara, CA, USA) was used to analyse the quality of the cDNA. By using the SOLiD Templated Bead Preparation guide, the whole-transcriptome libraries were utilised to generate SOLiD templated beads. Based on the parameters of the workflow analysis (WFA), bead quality was evaluated. The 50,625 paired-end procedure was used to sequence the samples, producing 75 nt + 35 nt (paired-end) + 5 nt (barcode) sequences. The program SETS parameters (SOLiD Experimental Tracking System) were used to measure the quality of the data.

The first set of whole-transcriptome and paired-end reads from the sequencing process were mapped using the Life Technologies mapping algorithm (http://www.lifetechnologies.com/, accessed on 15 May 2020), version 1.3, against the most recent version of the human genome (Version GRchr37/hg19). For both whole-transcriptome and paired-ends analysis, we employed the standard Bioscope settings of version 1.3. For both forward and reverse reads, the seed consisted in the first 25 nucleotides with a maximum of 2 mismatches permitted. The BAM/SAM format [61] was used to report aligned records. Using the Picard Tools software, version 1.83 (http://broadinstitute.github.io/picard/, accessed on 15 May 2020) [62], low-quality readings (Phred score < 10) were removed.

### 4.4. Statistical Methods

The mean ± standard deviation (SD) was used to express data for continuous variables, whereas percentage values were used for discrete variables. The Kolmogorov–Smirnov test was used to analyse the distribution of the data. The Mann–Whitney U test and the Student’s t-test were used to compare continuous variables with normal and non-normal distributions, respectively. For discrete variables, Fisher’s exact test was applied. To examine the relationships between variables having a normal distribution, Pearson’s correlation coefficients were computed.

The DESeq2 algorithm (version 3.4, retrieved on 15 May 2020) was utilised to evaluate the differential RNA expression between conditions [63]. To prevent false positives from being identified across the differential expression data, we regarded as differentially expressed those RNAs with a *p* value (*p* adj) adjusted by FDR < 0.05 [64]. Gene predictions were subsequently estimated using the cufflinks method [65], and the expression levels were calculated using the HTSeq software, version 0.5.4p3 [66]. Only unique readings were considered for the assessment of gene expression after the multimapped reads were removed using this approach. The differential expression between conditions was analysed using the edgeR technique, version 3.2.4 [67]. This approach is predicated on distinct normalisation procedures according to in-depth global samples, the composition of the CG and gene length. This approach uses a Poisson model to estimate the variance of the RNA-seq data for differential expressions during the differential expression process. The statistical analyses were conducted using the R statistic (version R-4.3.1) and SPSS software (version 20.0) for Windows (IBM SPSS Inc., Armonk, NY, USA). The STRING v12.0 program (accessible at https://string-db.org/, retrieved on 23 July 2024) was also used to explore protein–protein interactions. Databases, co-expression and experiments were among the criteria that were assessed.

## Figures and Tables

**Table 1:** Clinical characteristics of patients.

	mRNA-Seq	
	ICM (n = 13)	DCM (n = 13)
Gender male (%)	100	92
Age (years)	54 ± 8	51 ± 11
NYHA class	III–IV	III–IV
BMI (kg/m^2^)	27 ± 4	27 ± 5
Haemoglobin (g/dL)	14 ± 3	13 ± 3
Haematocrit (%)	41 ± 6	39 ± 7
Total cholesterol (mg/dL)	162 ± 41	147 ± 37
NT-proBNP (pg/mL)	3684 ± 2285	4071 ± 3584
Prior hypertension (%)	33	17
Prior smoking (%)	92 *	50
Diabetes mellitus (%)	42	17
Echocardiographic study		
LVEF (%)	25 ± 5 *	17 ± 8
LVESD (mm)	57 ± 8 ***	74 ± 10
LVEDD (mm)	65 ± 8 ***	81 ± 8
LVMI (g/cm^2^)	139 ± 26 ***	245 ± 64
Duration of disease (months)	45 ± 40	75 ± 68
Treatment (%)		
Renin–angiotensin system inhibitors	71	100
B-blokers	57	73
Aldosterone antagonists	100	91
Digoxin	43	64
Diuretics	71	100

ICM, ischaemic cardiomyopathy; DCM, dilated cardiomyopathy; NYHA, New York Heart Association; BMI, body mass index; LVEF, left ventricular ejection fraction; LVESD, left ventricular end-systolic diameter; LVEDD, left ventricular end-diastolic diameter; LVMI, left ventricular mass index; Duration of disease, from heart failure diagnosis until heart transplant. For differences between ICM and DCM in mRNA-seq study, * *p* < 0.05 and *** *p* < 0.001. Qualitative data are presented as percentages and quantitative data as mean values ± standard deviation.

## Data Availability

The data presented in this manuscript have been deposited in the NCBI’s Gene Expression Omnibus [69] (GEO) database and are accessible through the GEO series accession number GSE55296.

## Supplementary Materials

The following supporting information can be downloaded at: https://www.mdpi.com/article/10.3390/ijms25179590/s1.
