# Supplementary material for: Transcriptomic Alterations in Spliceosome Components in Advanced Heart Failure: Status of Cardiac-Specific Alternative Splicing Factors
Source: Int J Mol Sci. 2024 Sep 4;25(17):9590. doi: 10.3390/ijms25179590 (PMC11395552; doi:10.3390/ijms25179590)
Supplement: Supplementary file 1 [file ijms-25-09590-s001.zip › ijms-3164576-supplementary.pdf]

Supplemental Table S1. Components of the spliceosome.

N	Gene ID	Gene Name	Protein Name (UniProtKB)
**E complex**			
001	ENSG00000108654	*DDX5*	Probable ATP-dependent RNA helicase DDX5
002	ENSG00000007392	*LUC7L*	Putative RNA-binding protein Luc7-like 1
003	ENSG00000185246	*PRPF39*	Pre-mRNA-processing factor 39
004	ENSG00000196504	*PRPF40A*	Pre-mRNA-processing factor 40 homolog A
005	ENSG00000119707	*RBM25*	RNA-binding protein 25
006	ENSG00000168066	*SF1*	Splicing factor 1
007	ENSG00000104852	*SNRNP70*	U1 small nuclear ribonucleoprotein 70 kDa
008	ENSG00000077312	*SNRPA*	U1 small nuclear ribonucleoprotein A
009	ENSG00000124562	*SNRPC*	U1 small nuclear ribonucleoprotein C
010	ENSG00000125835	*SNRPB*	Small nuclear ribonucleoprotein-associated proteins B and B'
011	ENSG00000125870	*SNRPB2*	U2 small nuclear ribonucleoprotein B"
012	ENSG00000167088	*SNRPD1*	Small nuclear ribonucleoprotein Sm D1
013	ENSG00000125743	*SNRPD2*	Small nuclear ribonucleoprotein Sm D2
014	ENSG00000100028	*SNRPD3*	Small nuclear ribonucleoprotein Sm D3
015	ENSG00000182004	*SNRPE*	Small nuclear ribonucleoprotein E
016	ENSG00000139343	*SNRPF*	Small nuclear ribonucleoprotein F
017	ENSG00000143977	*SNRPG*	Small nuclear ribonucleoprotein G
018	ENSG00000113649	*TCERG1*	Transcription elongation regulator 1
**A complex**			
019	ENSG00000154473	*BUB3*	Mitotic checkpoint protein BUB3
020	ENSG00000060339	*CCAR1*	Cell division cycle and apoptosis regulator protein 1
021	ENSG00000008128	*CDK11A*	Cyclin-dependent kinase 11A
022	ENSG00000085872	*CHERP*	Calcium homeostasis endoplasmic reticulum protein
023	ENSG00000198563	*DDX39B*	Spliceosome RNA helicase DDX39B
024	ENSG00000145833	*DDX46*	Probable ATP-dependent RNA helicase DDX46
025	ENSG00000109606	*DHX15*	ATP-dependent RNA helicase DHX15
026	ENSG00000089280	*FUS*	RNA-binding protein FUS
027	ENSG00000197451	*HNRNPAB*	Heterogeneous nuclear ribonucleoprotein A/B
028	ENSG00000100410	*PHF5A*	PHD finger-like domain-containing protein 5A
029	ENSG00000110844	*PRPF40B*	Pre-mRNA-processing factor 40 homolog B
030	ENSG00000182872	*RBM10*	RNA-binding protein 10
031	ENSG00000134453	*RBM17*	Splicing factor 45
032	ENSG00000003756	*RBM5*	RNA-binding protein 5
033	ENSG00000099995	*SF3A1*	Splicing factor 3A subunit 1
034	ENSG00000104897	*SF3A2*	Splicing factor 3A subunit 2
035	ENSG00000183431	*SF3A3*	Splicing factor 3A subunit 3
036	ENSG00000115524	*SF3B1*	Splicing factor 3B subunit 1
037	ENSG00000115128	*SF3B14*	Splicing factor 3B subunit 6
038	ENSG00000087365	*SF3B2*	Splicing factor 3B subunit 2
039	ENSG00000189091	*SF3B3*	Splicing factor 3B subunit 3
040	ENSG00000143368	*SF3B4*	Splicing factor 3B subunit 4

041	ENSG00000169976	*SF3B5*	Splicing factor 3B subunit 5
042	ENSG00000119953	*SMNDC1*	Survival of motor neuron-related-splicing factor 30
043	ENSG00000131876	*SNRPA1*	U2 small nuclear ribonucleoprotein A'
044	ENSG00000105705	*SUGP1*	SURP and G-patch domain-containing protein 1
045	ENSG00000160201	*U2AF1*	Splicing factor U2AF 35 kDa subunit
046	ENSG00000063244	*U2AF2*	Splicing factor U2AF 65 kDa subunit
047	ENSG00000163714	*U2SURP*	U2 snRNP-associated SURP motif-containing protein
**B complex (B complex, Bact complex and B* complex)**			
048	ENSG00000021776	*AQR*	RNA helicase aquarius
049	ENSG00000116752	*BCAS2*	Pre-mRNA-splicing factor SPF27
050	ENSG00000106245	*BUD31*	Protein BUD31 homolog
052	ENSG00000160799	*CCDC12*	Coiled-coil domain-containing protein 12
053	ENSG00000168438	*CDC40*	Pre-Mrna-processing factor 17
056	ENSG00000096401	*CDC5L*	Cell division cycle 5-like protein
057	ENSG00000101343	*CRNKL1*	Crooked neck-like protein 1
058	ENSG00000132792	*CTNNBL1*	Beta-catenin-like protein 1
054	ENSG00000163510	*CWC22*	Pre-Mrna-splicing factor CWC22 homolog
055	ENSG00000153015	*CWC27*	Spliceosome-associated protein CWC27 homolog
059	ENSG00000174243	*DDX23*	Probable ATP-dependent RNA helicase DDX23
060	ENSG00000204560	*DHX16*	Pre-Mrna-splicing factor ATP-dependent RNA helicase DHX16
061	ENSG00000126698	*DNAJC8*	DnaJ homolog subfamily C member 8
062	ENSG00000141543	*EIF4A3*	Eukaryotic initiation factor 4A-III
063	ENSG00000108883	*EFTUD2*	116 kDa U5 small nuclear ribonucleoprotein component
064	ENSG00000076650	*GPATCH1*	G patch domain-containing protein 1
065	ENSG00000068394	*GPKOW*	G-patch domain and KOW motifs-containing protein
066	ENSG00000240682	*ISY1*	Pre-mRNA-splicing factor ISY1 homolog
067	ENSG00000171566	*PLRG1*	Pleiotropic regulator 1
068	ENSG00000084072	*PPIE*	Peptidyl-prolyl cis-trans isomerase E
069	ENSG00000171960	*PPIH*	Peptidyl-prolyl cis-trans isomerase H
072	ENSG00000137168	*PPIL1*	Peptidyl-prolyl cis-trans isomerase-like 1
070	ENSG00000100023	*PPIL2*	RING-type E3 ubiquitin-protein ligase PPIL2
071	ENSG00000143294	*PRCC*	Proline-rich protein PRCC
073	ENSG00000110107	*PRPF19*	Pre-mRNA-processing factor 19
074	ENSG00000117360	*PRPF3*	U4/U6 small nuclear ribonucleoprotein Prp3
075	ENSG00000105618	*PRPF31*	U4/U6 small nuclear ribonucleoprotein Prp31
076	ENSG00000134748	*PRPF38A*	Pre-mRNA-splicing factor 38A
077	ENSG00000134186	*PRPF38B*	Pre-mRNA-splicing factor 38B
078	ENSG00000136875	*PRPF4*	U4/U6 small nuclear ribonucleoprotein Prp4
079	ENSG00000101161	*PRPF6*	Pre-mRNA-processing factor 6
080	ENSG00000174231	*PRPF8*	Pre-Mrna-processing-splicing factor 8
081	ENSG00000125352	*RNF113A*	E3 ubiquitin-protein ligase RNF113A
082	ENSG00000144028	*SNRNP200*	U5 small nuclear ribonucleoprotein 200 kDa helicase

083	ENSG00000060688	*SNRNP40*	U5 small nuclear ribonucleoprotein 40 kDa protein
084	ENSG00000100138	*SNU13*	NHP2-like protein 1
085	ENSG00000100603	*SNW1*	SNW domain-containing protein 1
086	ENSG00000184281	*TSSC4*	U5 small nuclear ribonucleoprotein TSSC4
087	ENSG00000141759	*TXNL4A*	Thioredoxin-like protein 4A
088	ENSG00000076924	*XAB2*	Pre-Mrna-splicing factor SYF1
089	ENSG00000146007	*ZMAT2*	Zinc finger matrin-type protein 2
**C complex**			
090	ENSG00000105298	*CACTIN*	Splicing factor Cactin
091	ENSG00000185324	*CDK10*	Cyclin-dependent kinase 10
092	ENSG00000183258	*DDX41*	Probable ATP-dependent RNA helicase DDX41
093	ENSG00000100056	*DGCR14*	Splicing factor ESS-2 homolog
094	ENSG00000101452	*DHX35*	Probable ATP-dependent RNA helicase DHX35
095	ENSG00000067596	*DHX8*	ATP-dependent RNA helicase DHX8
096	ENSG00000105058	*FAM32A*	Protein FAM32A
097	ENSG00000071859	*FAM50A*	Protein FAM50A
098	ENSG00000148690	*FRA10AC1*	Protein FRA10AC1
099	ENSG00000092199	*HNRNPC*	Heterogeneous nuclear ribonucleoproteins C1/C2
100	ENSG00000109971	*HSPA8*	Heat shock cognate 71 kDa protein
101	ENSG00000105617	*LENG1*	Leukocyte receptor cluster member 1
102	ENSG00000162385	*MAGOH*	Protein mago nashi homolog
103	ENSG00000111196	*MAGOHB*	Protein mago nashi homolog 2
104	ENSG00000114503	*NCBP2*	Nuclear cap-binding protein subunit 2
105	ENSG00000142546	*NOSIP*	Nitric oxide synthase-interacting protein
106	ENSG00000138398	*PPIG*	Peptidyl-prolyl cis-trans isomerase G
107	ENSG00000240344	*PPIL3*	Peptidyl-prolyl cis-trans isomerase-like 3
108	ENSG00000113593	*PPWD1*	Peptidylprolyl isomerase domain and WD repeat-containing protein 1
109	ENSG00000165630	*PRPF18*	Pre-Mrna-splicing factor 18
110	ENSG00000143751	*SDE2*	Splicing regulator SDE2
111	ENSG00000164609	*SLU7*	Pre-Mrna-splicing factor SLU7
112	ENSG00000163877	*SNIP1*	Smad nuclear-interacting protein 1
113	ENSG00000128739	*SNRPN*	Small nuclear ribonucleoprotein-associated protein N
114	ENSG00000018610	*STEEP1*	STING ER exit protein
115	ENSG00000167978	*SRRM2*	Serine/arginine repetitive matrix protein 2
116	ENSG00000117614	*SYF2*	Pre-Mrna-splicing factor SYF2
117	ENSG00000105248	*YJU2*	Splicing factor YJU2
118	ENSG00000198783	*ZNF830*	Zinc finger protein 830

Supplemental Table S2. Cardiac-specific alternative splicing factors

N	Gene ID	Gene Name	Protein Name (UniProtKB)
001	ENSG00000149187	*CELF1*	CUGBP Elav-like family member 1
002	ENSG00000048740	*CELF2*	CUGBP Elav-like family member 2
003	ENSG00000159409	*CELF3*	CUGBP Elav-like family member 3
004	ENSG00000101489	*CELF4*	CUGBP Elav-like family member 4
005	ENSG00000161082	*CELF5*	CUGBP Elav-like family member 5
006	ENSG00000140488	*CELF6*	CUGBP Elav-like family member 6
007	ENSG00000135486	*HNRNPA1*	Heterogeneous nuclear ribonucleoprotein A1
008	ENSG00000153187	*HNRNPU*	Heterogeneous nuclear ribonucleoprotein U
009	ENSG00000131773	*KHDRBS3*	KH domain-containing, RNA-binding, signal transduction-associated protein 3
010	ENSG00000108848	*LUC7L3*	Luc7-like protein 3
011	ENSG00000152601	*MBNL1*	Muscleblind-like protein 1
012	ENSG00000139793	*MBNL2*	Muscleblind-like protein 2
013	ENSG00000076770	*MBNL3*	Muscleblind-like protein 3
014	ENSG00000139910	*NOVA1*	RNA-binding protein Nova-1
015	ENSG00000104967	*NOVA2*	RNA-binding protein Nova-2
016	ENSG00000169564	*PCBP1*	Poly(rC)-binding protein 1
017	ENSG00000197111	*PCBP2*	Poly(rC)-binding protein 2
018	ENSG00000181929	*PRKAG1*	5'-AMP-activated protein kinase subunit gamma-1
019	ENSG00000106617	*PRKAG2*	5'-AMP-activated protein kinase subunit gamma-2
020	ENSG00000115592	*PRKAG3*	5'-AMP-activated protein kinase subunit gamma-3
021	ENSG00000112531	*QKI*	KH domain-containing RNA-binding protein QKI
022	ENSG00000078328	*RBFOX1*	RNA binding protein fox-1 homolog 1
023	ENSG00000100320	*RBFOX2*	RNA binding protein fox-1 homolog 2
024	ENSG00000203867	*RBM20*	RNA-binding protein 20
025	ENSG00000086589	*RBM22*	Pre-Mrna-splicing factor RBM22
026	ENSG00000112183	*RBM24*	RNA-binding protein 24
027	ENSG00000132819	*RBM38*	RNA-binding protein 38
028	ENSG00000157110	*RBPMS*	RNA-binding protein with multiple splicing
029	ENSG00000166831	*RBPMS2*	RNA-binding protein with multiple splicing 2
030	ENSG00000136450	*SRSF1*	Serine/arginine-rich splicing factor 1
031	ENSG00000188529	*SRSF10*	Serine/arginine-rich splicing factor 10
032	ENSG00000116754	*SRSF11*	Serine/arginine-rich splicing factor 11
033	ENSG00000154548	*SRSF12*	Serine/arginine-rich splicing factor 12
034	ENSG00000161547	*SRSF2*	Serine/arginine-rich splicing factor 2
035	ENSG00000112081	*SRSF3*	Serine/arginine-rich splicing factor 3
036	ENSG00000116350	*SRSF4*	Serine/arginine-rich splicing factor 4
037	ENSG00000100650	*SRSF5*	Serine/arginine-rich splicing factor 5
038	ENSG00000124193	*SRSF6*	Serine/arginine-rich splicing factor 6
039	ENSG00000115875	*SRSF7*	Serine/arginine-rich splicing factor 7
040	ENSG00000111786	*SRSF9*	Serine/arginine-rich splicing factor 9
